# Whole-genome sequence analysis of Zika virus, amplified from urine of traveler from the Philippines

**DOI:** 10.1007/s11262-017-1500-9

**Published:** 2017-08-09

**Authors:** Se Hun Gu, Dong Hyun Song, Daesang Lee, Jeyoun Jang, Min Young Kim, Jaehun Jung, Koung In Woo, Mirang Kim, Woong Seog, Hong Sang Oh, Byung Seop Choi, Jong-Seong Ahn, Quehn Park, Seong Tae Jeong

**Affiliations:** 1 The 5th R&D Institute, Agency for Defense Development, Yuseong, P.O. Box 35, Daejeon, 34186 Republic of Korea; 2 Armed Forces Medical Research Institute, 90 Jaunro, Yuseong, Daejeon, 305-878 Republic of Korea; 3 Armed Forces Medical Command, 177 Saemaeul-ro, Bundang-gu, P.O. Box 100, Seongnam-Si, 463-040 Republic of Korea

**Keywords:** Zika virus, Next generation sequencing (NGS), Whole-genome sequence, South Korea, Philippines

## Abstract

Zika virus (ZIKV) (genus *Flavivirus*, family *Flaviviridae*) is an emerging pathogen associated with microcephaly and Guillain-Barré syndrome. The rapid spread of ZIKV disease in over 60 countries and the large numbers of travel-associated cases have caused worldwide concern. Thus, intensified surveillance of cases among immigrants and tourists from ZIKV-endemic areas is important for disease control and prevention. In this study, using Next Generation Sequencing, we reported the first whole-genome sequence of ZIKV strain AFMC-U, amplified from the urine of a traveler returning to Korea from the Philippines. Phylogenetic analysis showed geographic-specific clustering. Our results underscore the importance of examining urine in the diagnosis of ZIKV infection.

Zika virus (ZIKV), a single-stranded, positive-sense RNA virus belonging to the *Flavivirus* genus of the *Flaviviridae* family, is transmitted by mosquitoes of the *Aedes* species (*Ae. aegypti* and *Ae. albopictus*). ZIKV was first identified in a sentinel rhesus monkey in the Zika Forest in Uganda in 1947 [1–3]. Recently, ZIKV has become one of the most important mosquito-borne viruses, with outbreaks associated with microcephaly [4] and Guillain-Barré syndrome [5] in the Americas, Pacific, and Southeast Asia. As of January 2017, 17 ZIKV infection cases (13 male and 4 female) have been confirmed in Korea, according to the Korea Centers for Disease Control and Prevention (KCDC). All 17 cases have been related to travel to South America and Southeast Asia: one to Brazil (case #1) [6], seven to the Philippines (cases #2, 3, 5, 12, 13, 15, 17), four to Vietnam (cases #4, 9, 11, 16), one to the Dominican Republic (case #6), one to the Republic of Guatemala (case #7), one to Puerto Rico (case #8), and two to Thailand (cases #10, 14) (unpublished data from KCDC).

Here, we report the full-length genome sequence of ZIKV strain AFMC-U, amplified from the urine of a male recruit (case #3) in a Korean Army training center in the Republic of Korea, using next generation sequencing technology. In April 10–14, 2016, two brothers, ages 20 and 21 years (cases #2 and #3), returned to Korea from Boracay, Philippines. The younger brother (case #2) was hospitalized with flu-like symptoms and rash, and ZIKV infection was diagnosed in a urine sample, using Zika Virus Polyprotein gene genesig^®^ Standard Kit (Primerdesign Ltd, United Kingdom) (unpublished data from KCDC). Two weeks after returning, the older brother (case #3) joined the Korean Army, and although he was asymptomatic, serum, saliva and urine samples were collected. Total RNA was extracted from serum, saliva, and urine, using the QIAamp viral RNA mini kit (Qiagen, Hilden, Germany), and cDNA was prepared using the SuperScript III First-Strand Synthesis System (Invitrogen, San Diego, USA) and random hexamers. Oligo-nucleotide primer sequences for nested PCR were ZIKV-1F: 5′–AGTTGTTGATCTGTGTGAATCAGAC–3′ and ZIKV-637R: 5′–CATAGGGCATTCATAGCTCATGGT–3′, ZIKV-1F and ZIKV-397R: 5′–GCATTGATTATTCTCAGCATGGC–3′. Initial denaturation was 94 °C for 5 min, followed by 15 cycles of denaturation at 94 °C for 40 s, annealing at 50 °C for 40 s, elongation at 72 °C 1 min, then 25 cycles of denaturation at 94 °C for 40 s, annealing at 52 °C for 40 s and elongation at 72 °C for 1 min, in a. ProFlex™ PCR system (Applied Biosystems, Foster City, CA, USA). PCR products were purified by the QIAquick PCR purification Kit (Qiagen), and DNA sequencing was performed in both directions, using the Big-Dye terminator v3.1 cycle sequencing kit (Applied Biosystems) on an Applied Biosystems 3500 series Genetic Analyzer (Applied Biosystems). Both urine and saliva were positive, but serum was negative for ZIKV using RT-PCR.

To obtain the whole-genome sequence of ZIKV from urine and saliva of case #3 by next generation sequencing (NGS) technology, a library was prepared using TruSeq RNA Access Library Prep Kit (Illumina, San Diego, CA, USA) according to manufacturer’s instruction. The library sizes and molar concentrations were determined by the Bio-analyzer with the Agilent DNA 1000 Kit (Agilent Technologies, Inc., Santa Clara, CA, USA), and the libraries were quantified using the Library Quantification kit for Illumina sequencing platforms (KAPA Biosystems, Wilmington, MA, USA) and a Quantstudio 6 Flex Real-time PCR (Applied Biosystems). Deep sequencing of ZIKV from urine and saliva of case #3 were performed on a MiSeq benchtop sequencer (Illumina) using a MiSeq reagent kit version 2 (Illumina) with 2  ×  150 bp paired-end, according to manufacturer’s instructions. The 5′- and 3′-terminal sequences were filled by designing specific primers, using the conventional Sanger sequencing method and SMARTer^®^ RACE 5′/3′ Kit (Takara Bio Inc., Otsu, Japan). Total reads were qualified over Q20 score and trimmed for reference mapping (Reference sequence: NC_012532) and consensus sequences extraction using CLC Genomics Workbench version 7.5.2 (CLC Bio, Cambridge, MA, USA). Depth of coverage was calculated by the number of mapped reads (read length × number of reads matching to the reference/genome size of reference). NGS data from the urine sample (ZIKV strain AFMC-U) generated 1,012,451 reads (depth of coverage; 14,069.6) and saliva sample (ZIKV strain AFMC-S) generated 4791 reads (depth of coverage; 66.6) with a mean read length of 150 bases.

We obtained the complete-genome and partial-genome sequence of ZIKV from urine and saliva sample, respectively. The full-length genome sequence of ZIKV strain AFMC-U was 10,795 nucleotides (GenBank accession no. KY553111) with 51.4% G+C content and 107-(1 to 107) and 428-nucleotide (10,368 to 10,795) 5′- and 3′-untranslated region (UTR), respectively. A 9063 nucleotide of ZIKV strain AFMC-S (GenBank accession no. KY962729) and ZIKV strain AFMC-U were identical (Table 1; Fig. 1). Whole-genome sequence comparison between ZIKV strain AFMC-U and ZIKV/H.sapiens-tc/PHL/2012/CPC-0740 from the Philippines (GenBank accession no. KU681082) showed 98.6 and 99.6% sequence similarity at the nucleotide and amino acid level, respectively (Table 1). Phylogenetic analysis, based on the nucleotide sequences, generated by the neighbor-joining method with 1000 bootstrap replicates using MEGA 6 [7]. The phylogenetic tree showed that ZIKV strain AFMC-U belonged to the Asian lineage and was closely related to a ZIKV strain from the Philippines (Fig. 1) [8, 9].

**Table 1:** Nucleotide and amino acid sequence similarity (%) between ZIKV strain AFMC-U and representative flaviviruses

Virus	Isolate (strain)	Genome (bp)	Nucleotide (%)	Amimo acid (%)
Zika	AFMC-S	9063	100.0	100.0
Zika	H.sapiens-tc/PHL/2012/CPC-0740	10,807	98.6	99.6
Zika	H.sapiens-tc/KHM/2010/FSS13025	10,807	97.9	99.4
Zika	H.sapiens-tc/THA/2014/SV0127-14	10,807	97.6	99.3
Zika	SZ01/2016/China	10,272	92.7	99.4
Zika	SZ-WIV01	10,709	96.7	99.4
Zika	GZ01	10,272	92.6	99.4
Zika	GD01	10,574	95.4	99.4
Zika	PLCal_ZV	10,141	91.7	99.4
Zika	TS17-2016	10,806	97.7	99.4
Zika	H/PF/2013	10,807	97.8	99.5
Zika	PRVABC-59	10,807	97.6	99.4
Zika	P6-740	10,269	90.7	99.4
Zika	SSABR1	10,648	96.2	99.5
Zika	Rio-U1	10,795	97.5	99.4
Zika	MEX/InDRE/Sm/2016	10,617	95.6	99.3
Zika	Brazil-ZKV2015	10,793	97.5	99.4
Zika	Brazil/2016/INMI1	10,643	96.2	99.4
Zika	Haiti/1225/2014	10,807	97.6	99.4
Zika	ZikaSPH2015	10,676	96.4	99.3
Zika	ZIKV/H.sapiens/Brazil/PE243/2015	10,807	97.7	99.3
Zika	Paraiba_01	10,807	97.7	99.4
Zika	Natal RGN	10,808	97.6	99.4
Zika	Rio-S1	10,805	97.6	99.4
Zika	ArD128000	10,272	88.7	96.5
Zika	ARB13565	10,788	88.7	97.3
Zika	ArD158084	10,272	84.2	97.1
Zika	MR 766	10,794	89.0	96.6
Zika	MR766-NIID	10,807	89.0	96.6
Zika	MR 766	10,766	88.7	97.0
Spondweni	SM-6 V-1	10,290	65.1	74.8
West Nile	B956	11,038	56.5	56.9
Dengue 1	Hawaii	10,736	57.4	55.4
Dengue 2	D2/SG/CT38/2013	10,720	57.6	55.4
Dengue 3	H87	10,696	57.4	56.1
Dengue 4	H241	10,664	57.5	55.8

**Fig. 1:**
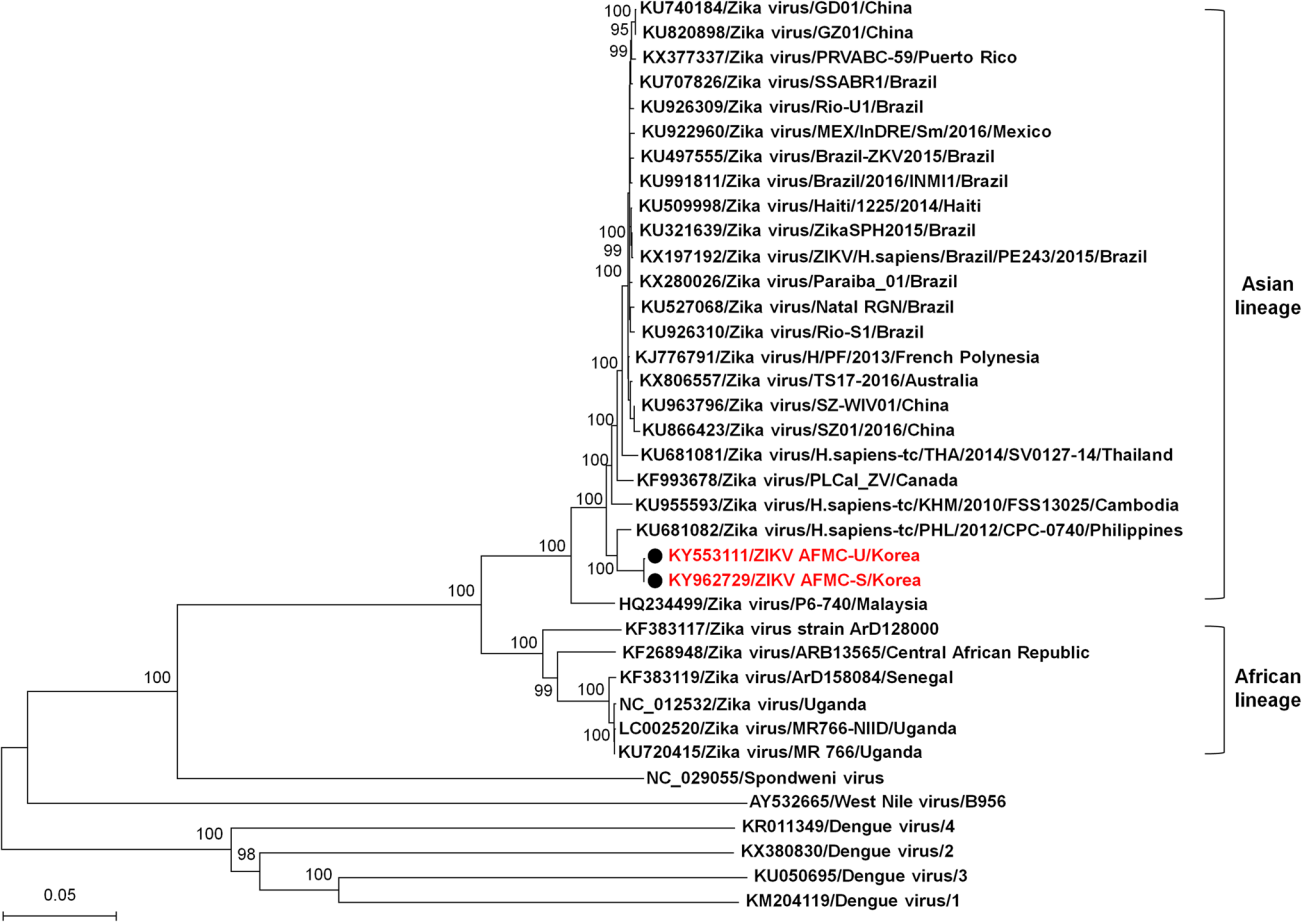
Phylogenetic analysis of the complete-genome sequences of Zika virus for a travel-associated case of Zika virus infection in a traveler returning to Korea from Boracay, Philippines, in April 2016. Phylogenetic tree was generated by the neighbor-joining method, using the Kimura 2-parameter model. *Scale bar* indicates number of base substitutions per site

This is the first report of the whole-genome sequence from a travel-associated case of ZIKV infection in the Republic of Korea. Our results underscore the importance of examining urine for ZIKV RNA. Although no cases of autochthonous transmission of ZIKV have been found in Korea, the presence of *Ae. albopictus* mosquitoes in rural and urban areas of Korea should heighten awareness of this possibility among physicians, as well as public health and vector control personnel.
